# Inflammation and serotonin deficiency in major depressive disorder: molecular docking of antidepressant and anti-inflammatory drugs to tryptophan and indoleamine 2,3-dioxygenases

**DOI:** 10.1042/BSR20220426

**Published:** 2022-05-27

**Authors:** Shazia Dawood, Samina Bano, Abdulla A.-B. Badawy

**Affiliations:** 1 Pharmacy and Allied Health Sciences, Iqra University, Karachi 7580, Pakistan; 2 Department of Biochemistry, Clinical Biochemistry and Psychopharmacology Research Unit, University of Karachi, Karachi 75270, Pakistan; 3 Formerly School of Health Sciences, Cardiff Metropolitan University, Western Avenue, Cardiff CF5 2YB, Wales, U.K.

**Keywords:** 3-dioxygenase, Glutamatergic activity, Kynurenine monooxygenase, Kynurenine pathway, Serotonin, Tryptophan 2

## Abstract

The roles of the kynurenine pathway (KP) of tryptophan (Trp) degradation in serotonin deficiency in major depressive disorder (MDD) and the associated inflammatory state are considered in the present study. Using molecular docking *in silico*, we demonstrate binding of antidepressants to the crystal structure of tryptophan 2,3-dioxygenase (TDO) but not to indoleamine 2,3-dioxygenase (IDO). TDO is inhibited by a wide range of antidepressant drugs. The rapidly acting antidepressant ketamine does not dock to either enzyme but may act by inhibiting kynurenine monooxygenase thereby antagonising glutamatergic activation to normalise serotonin function. Antidepressants with anti-inflammatory properties are unlikely to act by direct inhibition of IDO but may inhibit IDO induction by lowering levels of proinflammatory cytokines in immune-activated patients. Of six anti-inflammatory drugs tested, only salicylate docks strongly to TDO and apart from celecoxib, the other five dock to IDO. TDO inhibition remains the major common property of antidepressants and TDO induction the most likely mechanism of defective serotonin synthesis in MDD. TDO inhibition and increased free Trp availability by salicylate may underpin the antidepressant effect of aspirin and distinguish it from other nonsteroidal anti-inflammatory drugs. The controversial findings with IDO in MDD patients with an inflammatory state can be explained by IDO induction being overridden by changes in subsequent KP enzymes influencing glutamatergic function. The pathophysiology of MDD may be underpinned by the interaction of serotonergic and glutamatergic activities.

## Introduction

In accordance with the monoamine deficiency hypothesis of affective disorders, antidepressant drug development is based on inhibition of oxidation or reuptake of monoamine, particularly serotonin (5-hydroxytryptamine or 5-HT) and noradrenaline. More recently, inflammation has been implicated in major depressive disorder (MDD) and postulated to induce a serotonin deficiency by accelerating tryptophan (Trp) degradation along the kynurenine (Kyn) pathway (KP) by induction of the extrahepatic Trp-degrading enzyme indoleamine 2,3-dioxygenase (IDO1). The central serotonin pathway contributes very little (∼1%) to Trp degradation, whereas the KP contributes 95% [1,2]. Normally, the hepatic KP (rate-limited by Trp 2,3-dioxygenase or TDO, formerly Trp pyrrolase; EC 1.13.11.11) contributes 90%, whereas the extrahepatic pathway (rate-limited by IDO1; EC 1.13.11.17) contributes the remaining 5%, but, after immune activation, the extrahepatic pathway assumes a greater quantitative significance. The importance of liver TDO in control of Trp availability for cerebral serotonin synthesis is well established and evidenced more recently from studies demonstrating that deletion of the mouse TDO2 gene increases plasma [Trp] by 9.3- to 12.7-fold [3,4] and brain [Trp] by 10.6-fold in association with increased serotonin synthesis [5]. Deletion of the IDO1 or IDO2 genes makes little difference to Trp availability to the brain or to central serotonin synthesis [5]. Brain [Trp] is the major determinant of serotonin synthesis, because Trp hydroxylase (TPH2), the rate-limiting enzyme, is partially (≤50%) saturated with its Trp substrate [6], hence the suggestion of Trp as the key to boosting brain serotonin synthesis in MDD [7].

The KP produces a range of biologically active metabolites. Of particular importance to MDD are the antagonist kynurenic acid (KA) and agonist quinolinic acid (QA) of the N-methyl-D-aspartate (NMDA) type of receptors of the excitatory amino acid glutamate, and immunomodulation by these two metabolites and by 3-hydroxykynurenine (3-HK), 3-hydroxyanthranilic acid (3-HAA) picolinic acid (PA) and xanthurenic acid (XA). Overproduction of these metabolites can be deleterious to neuronal function and immune activity. Thus, modulation of NMDA receptor function can influence the state of neuronal excitability, which appears to be determined by the balance between KA and QA [8], and it is of interest that altered NMDA receptor function through modulation of KP activity has been implicated in the rapid antidepressant action of ketamine [9]. On the other hand, elevation of 3-HK, 3-HAA and QA induces immunosuppression by undermining T-cell function [1]. Increased production of Kyn metabolites, however, is not determined solely by TDO or IDO activity but also occurs through increased flux of plasma free (non-albumin-bound) Trp down the KP [10]. Also important is that KP metabolite levels can still be altered by changes in pathway enzyme activities in the absence of TDO/IDO induction or increased flux of free Trp.

The central serotonin deficiency in MDD is due to excessive peripheral and/or central Trp degradation via the KP. Accelerated Trp degradation in MDD is suggested by the widely reported decrease in circulating [Trp] [7] and from measurement of breath ^13^CO_2_ following administration of 1-^13^C-Trp [11]. The serotonin deficiency may involve liver TDO induction by cortisol, whose levels are elevated in at least 50% of depressed patients, or its activation by Trp-mediated catecholamine-related mechanisms as part of the stress response [7]. That immune activation acting through IDO induction in the periphery or the brain can also induce a central serotonin deficiency is exemplified by the incidence of depression in hepatitis C virus (HCV) patients treated with the IDO inducer interferon-α (IFN-α) (see [12] and references cited therein). The concept of IDO induction leading to serotonin deficiency in MDD may have arisen in part by extrapolation from the above and other studies with IFN-α. In HCV patients and related experimental models, the KP is already compromised by the virus and adding IFN-α simply exaggerates the KP changes and effects. Thus, HCV before initiation of antiviral therapy decreases plasma [Trp] by ∼9–24% and increases plasma [Kyn] by ∼18–24% [13,14]. In the mouse hepatitis virus model, the simultaneous elevation of Trp and Kyn levels in liver [15] is consistent with increased flux of plasma free (non-albumin-bound) Trp [16].

Although the immune status in MDD has received much attention [17], it is not clearly understood or well-defined. Not all MDD patients have an activated immune system and, when present, inflammation is of a mild and/or transient nature and its reversal is not associated with clinical outcome [9,17]. These features, compounded by heterogeneity of the disorder, associated comorbidities and biological factors, such as hypothalamic–pituitary–adrenal (HPA) axis activity and the immune status, illustrate the need for further studies. It is therefore reasonable to assume that immune activation in MDD, if strong enough, can impact Trp metabolism negatively through IDO induction, but that other mechanisms not involving IDO or overriding its effects must also operate in this disorder.

Whereas a range of mechanisms of actions of antidepressants exist, none of which is common to the majority of the drugs, except their ability to inhibit liver TDO activity both *in vitro* and after administration [7]. Antidepressants inhibit TDO activity significantly in doses as little as 0.5 mg/kg body wt [7]. Whereas much information is available on liver TDO inhibition by antidepressants, little is known about their potential effects on IDO activity, possibly because IDO activity is hardly detectable in the absence of immune activation and, even when IDO is highly expressed, investigators use the plasma [Kyn]/[Trp] ratio as an indirect measure of its activity, though this ratio is not specific to IDO, as it can also reflect changes in TDO activity, flux of plasma free Trp and activities of KP enzymes such as Kyn monooxygenase (KMO), and kynureninase [10]. The technique of molecular docking *in silico* is a useful tool for screening potential inhibitors of target proteins [18,19], but can also be applied to confirm binding of known inhibitors, e.g., with the TDO inhibitors 680C91 and LM10 as potential cancer therapies [20]. As far as we could ascertain, ours is the first group to use this technique to demonstrate binding (docking) of established antidepressant drugs to the TDO crystal structure. We showed that all eight antidepressants tested fitted well in the TDO active site with docking scores (kcal/mol) ranging from −109.796 (sertraline) to −139.706 (paroxetine) [21]. In the present paper, we report similar results with the TDO inhibitors tianeptine and venlafaxine and the failure of the non-antidepressant pargyline and the anti-inflammatory mefenamic acid, both of which do not inhibit TDO, to dock to it. Venlafaxine was chosen as the first joint serotonin-noradrenaline reuptake inhibitor (SNRI) to be shown to inhibit TDO. Tianeptine was chosen because its mode of action is still unclarified and, although it stimulates, rather than inhibits, serotonin reuptake, it is a clinically effective antidepressant, even in patients with poor response to serotonin-specific reuptake inhibitor (SSRI) monotherapy [22]. We also examined docking to IDO of the above 2 and 4 more antidepressants previously shown [21] to dock to TDO and compared their docking behaviours toward TDO and IDO with those of 6 nonsteroidal anti-inflammatory drugs (NSAIDs) not examined before. We further explored in some detail docking and other effects of aspirin, the only NSAID to dock to and inhibit TDO activity via its active metabolite salicylic acid, and ketamine, as its mode of action appears to be a novel one, modulating glutamatergic activity to restore serotonin homeostasis.

## Materials and methods

### Drugs and chemicals

Haematin hydrochloride and *L*-tryptophan were purchased from the Sigma chemical Co (St, Louis, Mo, U.S.A.) The antidepressants tianeptine sodium and venlafaxine were gift from Servier, France and Genetics Pharmaceutical, U.K., respectively. Pargyline and mefenamic acid were also gifts from Abbott Laboratories and Park Davis & Co, respectively. All other chemicals and reagents were from Sigma and/or BDH Chemicals (both of Poole, Dorset, U.K.) and were of the purest commercially available grades.

### Computational studies

Docking to TDO of antidepressant and anti-inflammatory drugs and the non-antidepressant pargyline was performed using the Molegro virtual Docker (MVD) software as described previously [21]. Re-ranking was performed to improve accuracy. When re-ranked, 10 independent docking runs resulted in 10 solutions. The structures of the tested drugs (Supplementary Figure S1) were imported into the MVD work space in SDF format. All H atoms were added and important valence checked by using the utilities in MVD. The crystal structure of TDO from *Xanthomonas campestris* in complex with ferrous heme and Trp (Northeast Structural Genomics Target XcR13: PDB ID: 2NW8), was taken from the protein data bank (http://www.rcsb.org/pdb) [23] into MVD work region at reasonable resolution levels (≤2.8 Å). In each docking run, high-quality orientation was analysed and hydrogen bonds identified and labeled. A linear combination of E-inter and E-intra bonding ligand energy was inspected and analysed using an MVD score. The TDO PDB protein structure was not energy minimized with the heavy atoms constrained before docking, because minimizing energy before docking is not always necessary or convenient and may change the protein structure and therefore affect the outcome of desired simulation.

**Figure 1:**
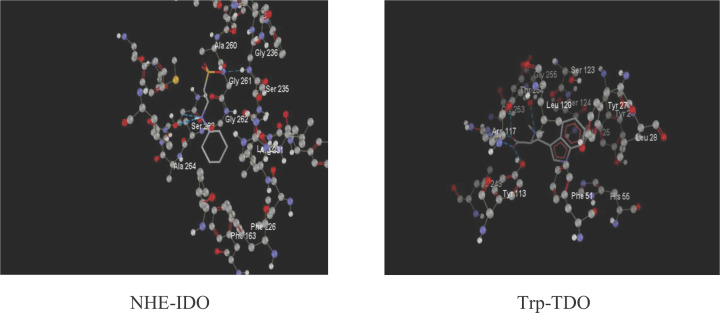
Ligand binding to the crystal structures of tryptophan and indoleamine 2,3-dioxygenases NHE is an indole compound, 2-[N-cyclohexylamino] ethanesulfonic acid, that mimics IDO substrates sufficiently to demonstrate substrate binding to the enzyme. Its MolDock and rerank scores are −89.5365 and -68.5943, with an RMSD of 1.85566 and H bond energy of −5.4088. The corresponding values for the Trp-TDO binding are −139.546, −99.9363, 3.46611 and −3.16738. For binding of NHE to IDO, the amino acids at the active site were Ser 263, Gly 262, Ala 264,260, Gly 236, Ser 235 and Gly 261 and the ligand binding amino acids were Ser 263, Gly 262 and Ala 264. For binding of Trp to TDO, the corresponding amino acids were Arg 117, Gly 253, Thr 254, Tyr 113, Leu 120, Ser 124 and Ser 123, and Arg 117, Thr 254 and Tyr 113.

Docking to IDO was also performed with the same drugs and metabolites. The IDO crystal structure selected for this study (PDB ID: 2D0T), that of the 4-phenylimidazole bound form of human indoleamine 2,3-dioxygenase (PDB doi:10.2210/pdb2D0T/pdb) [24], was downloaded into the MVD workspace from the protein data bank (http://www.rcsb.org/pdb) at reasonable resolution criteria (≤2.8 Å). Binding of Trp to TDO and of NHE (2-[N-cyclohexylamino] ethanesulfonic acid) to IDO is illustrated in Figure 1.

The MolDock scoring function used by MVD is derived from the PLP scoring functions originally proposed by Gehlhaar et al. (1995, 1998) and extended later by Yang and Chen (2004). The 10 solutions obtained from the 10 independent docking runs were re-ranked, in order to further increase the docking accuracy, by using a more complex scoring function. In MVD, along with the docking scoring function terms, a Lennard Jones 12-6 potential (Morris et al., 1998) (for these references, see [18,21]) and sp2 -sp2 torsion terms were also used. On the basis of pilot docking studies, the MolDock rerank scores were selected for ranking the inhibitor poses. The structure of all ligand were downloaded from pubchem and imported to the MVD workspace in ‘sdf’ format. In order to make accurate predictions, it is important that the imported structures have been properly prepared, that is, the atom connectivity and bond orders are correct and partial atomic charges are assigned. PDB files often have poor or missing assignment of explicit hydrogens, and the PDB file format cannot accommodate bond order information. All necessary valency checks and H atom addition were thus performed using the utilities provided in MVD. The binding site specifies the region of interest where the docking procedure will look for promising poses (ligand conformations). MolDock automatically identifies potential binding sites (also referred as cavities or active sites) by using its cavity detection algorithm. The cavities within a 30 × 30 × 30 Å^3^ cube centered at the experimentally known ligand position were used.

### Preparation of liver homogenates and determination of tryptophan 2,3-dioxygenase activity

Locally bred male Albino Wistar rats weighing 180–290 g were housed five per cage at 22 ± 1°C and maintained on lab chow and drinking water. Rats were killed between 13:00 and 14:00 h and livers were removed within 10 s and perfused *in situ* with ice-cold saline (to remove the blood source of heme) before freezing in liquid nitrogen until analysis. Two-gram portions of the frozen livers were homogenized in 13 ml of ice-cold 0.14 M KCl-0.0025 M NaOH for 1min in ice-packed tubes using an Ultraturrax homogenizer and were used within 4–9 min of preparation. TDO activity was determined in liver homogenates by quantifying the formation of kynurenine from *L*-tryptophan in the absence (holoenzyme activity) or presence (total enzyme activity) of added hematin as described previously [25], with the apoenzyme activity calculated by difference.

A 15 ml portion of the homogenates was added to a mixture of 5 ml of 0.03 M *L*-Trp, 15 ml of 0.2 M sodium phosphate buffer, pH 7.0, and 25 ml of water at 0°C. For the total TDO activity, haematin hydrochloride was dissolved in 0.1 M NaOH and 0.l ml was included in the overall mixture to give a final concentration (2 µM) that was previously shown to be optimal for enzyme activation [26]. Samples (3 ml) of the assay mixture were incubated at 37°C for 0, 15, 30, 45, 60 and 75 min with shaking (120 oscillations/min) in stoppered 25 ml conical flasks in an atmosphere of O_2_. The reaction was stopped at each time intervals by the addition of 2 mL of 0.9 M trichloroacetic acid; the flasks and contents were shaken for a further 2 min and then filtered on Whatman no. 1 filter paper. To a measured portion of the filtrate (2.5 ml) was added 1.5 ml of 0.6 M NaOH and the kynurenine present was determined by measuring the *E*_365_ with a Unicam SP.500 spectrophotometer and by using *ε* = 4540 L.mol^−1.^cm^−1^. A lag phase persisted for the first 30–45min of incubation and enzyme activity was calculated from the increase in the *E*_365_ during the linear phase. For an extinction range of 0.1–1.0, the line representing the linear phase exactly covered points at three to four consecutive time intervals. The validity of this visual method was mathematically validated [25]. Drugs were dissolved in deionized water, neutralized to pH 7.3 with 1 M HCl and added at different concentrations (10 µM to 1 mM).

### Statistical analysis

Data was analysed using unpaired *t* test. A probability *P* level of <0.05 was considered significant.

## Results

### Docking of drugs to TDO

Results of the docking of drugs to TDO and IDO are summarized in Table 1, detailed in Tables 2 and 3 and illustrated in Supplementary Figures S2–S6. Perfect fits of the various ligands to TDO and IDO is illustrated by all ligands having root mean square deviation (RMSD) values of zero, except indomethacin which had a value of 1. As shown in Table 2 and Supplementary Figure S2, both venlafaxine and tianeptine docked strongly to TDO, whereas the non-antidepressant pargyline did not. We showed previously [21] that the other four antidepressants listed in Tables 1 and 2 also docked strongly to TDO. The rapidly acting antidepressant ketamine, however, did not dock to TDO (Table 2 and Supplementary Figure S6). By contrast, only sodium salicylate among six NSAIDs docked to TDO (Table 2 and Supplementary Figure S3). Three diclofenac metabolites that mediate some of the pharmacological effects of their parent compound did not dock to TDO (Table 2 and Supplementary Figure S6).

**Table 1:** Summary of docking of antidepressant and anti-inflammatory drugs to TDO and IDO

Antidepressant drugs docking to			Anti-inflammatory drugs docking to		
Drug	TDO	IDO	Drug	TDO	IDO
Venlafaxine	+	-	Aspirin	-	+
Tianeptine	+	-	Salicylate	+	+
Amoxepine	+	-	Mefenamic acid	-	+
Paroxetine	+	-	Indomethacin	-	+
Moclobemide	+	-	Celecoxib	-	-
Fluvoxamine	+	-	Diclofenac	-	+
Ketamine	-	-	3-Hydroxy	-	-
Pargyline^*^	-		4-Hydroxy	-	-
			4,5-Dihydroxy	-	-

* Does not inhibit TDO nor act as antidepressant.

**Table 2:** Molecular docking to TDO, re-ranking scores and hydrogen bond energy of antidepressants, pargyline and anti-inflammatory drugs

Drugs	Docking score (Kcal/mol)	Re-ranking score	Log *P*	M Wt	Torsion number	HBA	HBD	HBE
Antidepressants								
Venlafaxine	−93.3835	−62.4479	2.74	277.4	5	3	1	−2.34707
Tianeptine	−68.980	−51.092	2.87	437	8	5	3	−3.26887
Amoxepine*	−120.086	−87.6386	3.08	288	1	3	1	−0.748501
Paroxetine*	−139.706	−110.963	3.15	313	4	4	1	−3.31147
Moclobemide*	−121.275	−86.5591	1.45	244	4	3	1	−4.06079
Fluvoxamine*	−133.451	−90.2595	2.80	315	9	4	2	−4.60011
R Ketamine	8045.94	79.6472	3.1	237.7	2	2	1	−0.211
Ketamine HCl	6076	164.647	3.1	237.7	2	0	0	0
Non-antidepressant								
Pargyline	1952.76	15.3495	−1.43	159.22	4	0	0	0
Anti-inflammatory								
Aspirin	1989.23	23.8794	1.25	179.14	3	3	1	−2.9052
Salicylate	−82.0858	−66.7095	−1.43	138	0	3	1	−12.385
Mefenamic acid	5977.33	27.5291	3.84	240	3	2	1	−2.56848
Indomethacin	4043.1	187.623	3.53	356.7	4	4	1	−1.925
Celecoxib	9974.6	54.0047	3.5	381.4	4	7	1	−1.487
Diclofenac	4011.2	52.289	4.14	296.14	4	2	0	−2.182
3-Hydroxy	3010.57	63.0403	4	312.14	4	4	4	−5.4734
4-hydroxy	1992.86	44.9216	3.7	312.14	4	4	3	−6.0116
3,4-Dihydroxy	4011.2	52.289	4.14	328.15	4	2	0	−5.47

Abbreviations: HBA, hydrogen bond acceptor; HBD, hydrogen bond donor; HBE, hydrogen bond energy (kcal/mol). The * denotes data from our previous publication [21] [Dawood, S., Zarina, S. and Bano, S. (2014) Docking studies of antidepressants against single crystal structure of tryptophan 2,3-dioxygenase using Molegro Virtual Docker software. *Pakistan J. Pharmaceut. Sci.* **27**, 1529–1539].

**Table 3:** Molecular docking to IDO, re-ranking scores and hydrogen bond energy of antidepressant and anti-inflammatory drugs

Drugs	Docking score (Kcal/mol)	Re-ranking score	Log *P*	M Wt	Torsion number	HBA	HBD	HBE
Antidepressants								
Venlafaxine	8082.72	147.007	2.74	277.4	5	3	1	0
Tianeptine	6083	199	2.87	437	8	5	3	−2.79
Amoxepine	7009.3	92.185	3.08	313.7	1	3	1	−2.5
Paroxetine	5965.4	43.924	3.15	329.3	4	4	1	−0.503
Moclobemide	4940	−30.27	1.45	268.07	4	3	1	−1.3
Fluvoxamine	4996.81	14.308	2.8	318.33	9	4	2	−1.98
R Ketamine	9074.58	74.9601	3.1	237.72	2	0	0	0
Ketamine HCl	8081.89	90.2596	3.1	237.72	2	0	0	0
Anti-inflammatory								
Aspirin	−110.253	−95.3902	1.24	179.14	3	3	1	−1.5
Salicylate	−66.6195	−52.8284	-1.43	138	0	3	1	−2.5
Mefenamic acid	−115.731	−95.9268	3.84	240	2	2	1	−1.5
Indomethacin	−161.647	−118.1	3.53	356.7	4	4	1	−4.16
Celecoxib	8011.03	77.4999	3.5	381.4	4	7	1	−0.419
Diclofenac	−147.324	−115.0	4.14	296.14	5	2	0	0
3-Hydroxy	3074.14	107.33	4	312.14	4	4	3	−1.606
4-Hydroxy	2025.25	50.9426	3.7	312.14	4	4	3	−6.8767
4,5-Dihydroxy	3047.71	82.6072	4.14	328.15	4	2	0	−6.47

Abbreviations: HBA, hydrogen bond acceptor; HBD, hydrogen bond donor; HBE, hydrogen bond energy (kcal/mol)

### Docking of drugs to IDO

None of the six antidepressants or ketamine docked to IDO (Table 3 and Supplementary Figures S4 and S6). Of the 6 NSAIDs tested, only celecoxib did not dock to IDO (Table 3 and Supplementary Figure S5). The three hydroxylated diclofenac metabolites also did not dock to IDO (Table 3 and Supplementary Figure S6).

### TDO inhibition by venlafaxine and tianeptine

That TDO activity is inhibited directly *in vitro* by venlafaxine and tianeptine is shown in Table 4. In line with other antidepressants, both drugs inhibit the conjugation of the apoenzyme with its heme cofactor. Thus, inhibition is specific to the apoenzyme, with tianeptine causing 58, 75, 75 and 75% inhibition and venlafaxine causing 82, 73, 91 and 64% inhibition at concentrations of 10 µM and 0.1, 0.5 and 1 mM, respectively. The non-antidepressant pargyline and the NSAID mefenamic acid have previously been shown [27] not to inhibit TDO activity or to elevate brain [Trp] following their acute administration to rats.

**Table 4:** Inhibition of rat liver tryptophan 2,3 dioxygenase activity *in vitro* by tianeptine and venlafaxine

Drug	Concentration	Kynurenine formed (µmol/g wet wt of liver/h)		
		Holoenzyme	Total enzyme	Apoenzyme
Tianeptine	0	1.2 ± 0.08	2.4 ± 0.18	1.2 ± 0.11
	10 µM	1.2 ± 0.10	1.7 ± 0.14*	0.5 ± 0.12**
	0.1 mM	1.2 ± 0.11	1.5 ± 0.07**	0.3 ± 0.04***
	0.5 mM	1.1 ± 0.06	1.4 ± 0.17**	0.3 ± 0.15**
	1 mM	1.0 ± 0.06	1.3 ± 0.13**	0.3 ± 0.12*
Venlafaxine	0	2.0 ± 0.32	3.1 ± 0.24	1.1 ± 0.17
	10 µM	2.2 ± 0.27	2.4 ± 0.26	0.2 ± 0.18*
	0.1 mM	2.2 ± 0.27	2.5 ± 0.14	0.3 ± 0.13*
	0.5 mM	2.3 ± 0.30	2.4 ± 0.32	0.1 ± 0.06**
	1 mM	1.9 ± 0.30	2.3 ± 0.19*	0.4 ± 0.12**

TDO activity was determined as described in the Materials and Methods either in the absence (holoenzyme activity) or presence (total enzyme activity) of added (2 µM) hematin. The apoenzyme activity was obtained by difference. Values are means ± SEM for at least three separate experiments per drug. The significance of the differences is indicated as follows: *P<0.05; **P<0.01; ***P<0.001.

### Effects of sodium salicylate on tryptophan metabolism

As the active aspirin metabolite and only NSAID to dock to TDO, sodium salicylate exerts other important effects on Trp metabolism [28,29]. It inhibits TDO (Trp pyrrolase) activity at doses of ≥0.5 mg/kg and elevates plasma free [Trp] following displacement of the albumin-bound amino acid at doses of ≥2.5 mg/kg. However, a large dose of salicylate (400 mg/kg) exerts a biphasic effect on TDO activity, a major Trp-mediated activation following the initial inhibition [28].

## Discussion

The present molecular docking results have demonstrated the binding of tianeptine and venlafaxine to the active site of the Trp 2,3-dioxygenase (TDO) crystal structure (Table 2 and Supplementary Figure S2). Their direct inhibition of TDO activity *in vitro* has also been demonstrated (Table 4). We previously found that other TDO-inhibitory antidepressants (a monoamine oxidase inhibitor, a tricyclic and 6 SSRIs) also dock strongly to TDO [21]. We also found in the present study that two drugs devoid of TDO inhibition (pargyline and mefenamic acid) do not dock to TDO. Taken together, our present and previous study demonstrate the validity and usefulness molecular docking for confirming the binding of TDO inhibitory antidepressants to the enzyme and absence of binding by non-inhibitors, and for identifying potential TDO inhibitors for use as antidepressants in future drug developmental studies. The present study is also the first to demonstrate that sodium salicylate, the active aspirin metabolite, is the only one of six established NSAIDs to dock to TDO and that of these six drugs, only celecoxib does not dock to IDO. Ketamine is the only antidepressant tested that failed to dock to TDO or IDO (Tables 2 and 3; Supplementary Figure S6). The mechanism of the antidepressant action of this rapidly acting drug is currently under investigation [9,30]. Its failure to bind to TDO or IDO, and thus unlikely to inhibit either enzyme, suggests that it may not enhance serotonin synthesis. Ketamine is also unlikely to exert its anti-inflammatory effects in humans via IDO inhibition. Potential mechanisms of the ketamine effects on the KP in MDD are discussed below.

### Inflammation and the kynurenine pathway in MDD

**Figure 2:**
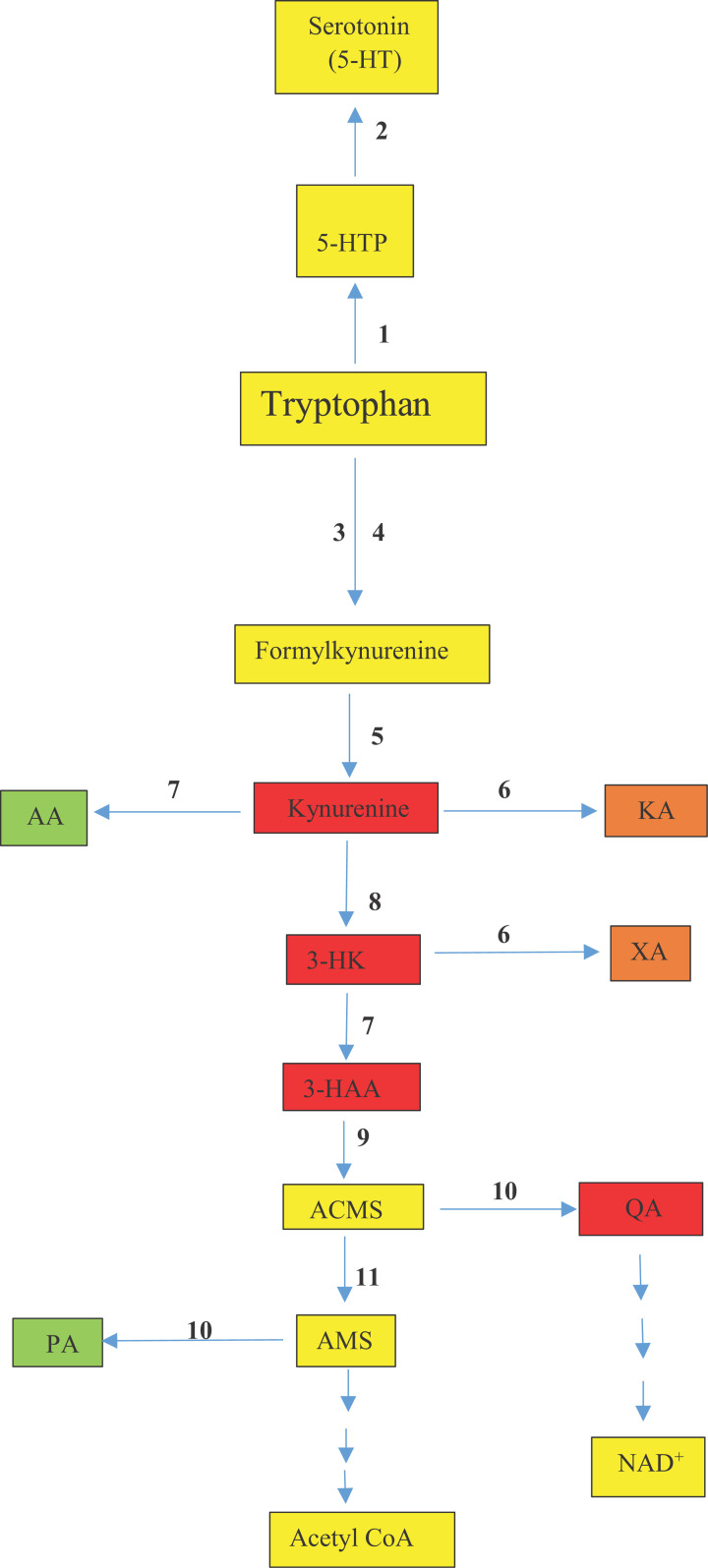
The kynurenine and serotonin pathways of tryptophan degradation Enzymes are numbered as follows: 1, tryptophan hydroxylase; 2, aromatic *L*-amino acid decarboxylase; 3, tryptophan 2,3-dioxygenase; 4, indoleamine 2,3-dioxygenase; 5, aryl hydrocarbon formamidase; 6, kynurenine aminotransferase; 7, kynureninase; 8, kynurenine monooxygenase; 9, 3-hydroxyanthranilic acid 3,4-dioxygenase; 10, non-enzymic cyclization; 11, 2-amino-3-carboxymuconate-6-semialdehyde decarboxylase or picolinate carboxylase. Abbreviations: AA, anthranilic acid; ACMS, 2-amino-3-carboxymuconate-6-semialdehyde; KMO (kynurenine monooxygenase); 3-HAA, 3-hydroxyanthranilic acid; 3-HK, 3-hydroxykynurenine; 5-HT, 5-hydroxytryptamine; 5-HTP, 5-hydroxytryptophan; KA, kynurenic acid; NAD^+^, oxidised nicotinamide-adenine dinucleotide; PA, picolinic acid; QA, quinolinic acid; XA, xanthurenic acid. Colour code: red, proinflammatory; green, anti-inflammatory; amber, mixed-type; yellow, normal metabolite.

Because inflammation has been proposed to induce the serotonin deficiency in MDD by IDO induction, a brief discussion of changes in IDO-inducing proinflammatory cytokines in MDD is useful. Elevation of cytokine levels is not universal among MDD patients [9,17] and the notable elevation of interleukins IL-1β, IL-6, IL-12, IL-18 and tumor-necrosis factor TNF-α in some patients does not appear to be associated with an inflamed subgroup but rather to a right shift of the immune marker distribution [31]. Changes in the [Kyn]/[Trp] ratio used to express IDO activity are contradictory, partly because of the non-specificity of this test, as an increase in this ratio is not limited to IDO induction and could be masked by changes in subsequent KP enzymes (Figure 2). For example, the low [Kyn] and [KA] levels in unmedicated patients [32] could be the result of elevated Kyn monooxygenase (KMO) activity depleting [Kyn], thereby depriving KAT of its substrate. IDO induction, however, does not play a role in MDD symptoms or response to antidepressant therapy [33,34]. Thus, it appears that IDO induction, if it occurs in patients with an inflamed state, is overridden by potential overexpression of KMO by IL-1β and IFN-γ [35–38]. The increases in [3-HAA] and [AA] by IFN-γ [38] suggest also activation of kynureninase, which has already been reported [35,36,39]. Many antidepressant drugs are postulated to possess anti-inflammatory properties. Their efficacy is, however, unlikely to be due to direct IDO inhibition, but, rather, to inhibition of KMO activation by proinflammatory cytokines, as has been demonstrated with ketamine [9] and escitalopram [40]. As KMO activation can result in increased production of the excitotoxic QA at the expense of the cytoprotective KA, future studies should focus on KMO as a potential determinant of neurological dysfunction in MDD.

Neuronal activity, however, can influence and be influenced by serotonin function in MDD. The mutual interactions between serotonergic and glutamatergic activities are well established [41–45] and can be viewed simplistically as low serotonin losing control over glutamate neurotransmission and inhibition of the latter facilitating serotonin function.

### The status of anti-inflammatory drugs in MDD therapy

With established NSAIDs, only aspirin possesses antidepressant properties in humans and experimental models of depression [46–54]. These favorable outcomes occur in studies performed over a relatively short- to medium-term period with daily doses of aspirin equivalent to 2.3 or 14.3 mg/kg body weight for a 70 kg adult. By contrast, long-term aspirin use, even in small doses, may be associated with increased depression [55,56]. These apparently contradictory clinical effects of aspirin can be explained by several mechanisms based on modulation of Trp metabolism and immune function by aspirin. The antidepressant effects of aspirin at therapeutic dose levels may involve TDO inhibition, but not direct IDO inhibition, and also Trp displacement from albumin-binding sites by its major hydrolytic metabolite salicylic acid. Thus, salicylate at doses as little as 0.5 and 1.0 mg/kg body weight inhibits rat liver TDO (Trp pyrrolase) activity without altering the binding of Trp to serum albumin, whereas larger doses continue to inhibit TDO activity, but, additionally, release Trp from albumin binding sites [29]. Both aspirin and salicylate do not inhibit IDO activity directly, even-though both dock to IDO (Table 3). They are, however, only partially effective in blocking IDO induction by IDO inducing treatments at a concentration (5 mM) [57] unlikely to be reached in therapeutic settings. Aspirin may, however, act in MDD patients with an inflamed state by decreasing IDO induction by interferon-γ [57], possibly by influencing cyclooxygenase 2 activity [58,59]. The incidence of depression with long-term use of even moderate doses of aspirin may involve activation and stabilization of TDO by the continued increase in Trp availability to the liver [25] leading to decreased serotonin synthesis. Increased TDO activity under these conditions leads to enhanced KP activity, as occurs in rheumatoid arthritis patients receiving long-term aspirin therapy exhibiting increased urinary excretion of Kyn, XA and 3-HAA [60]. We compared urinary Kyn levels within the control group in the study by Spiera [60] between those receiving aspirin and those not on aspirin and found that the former subgroup excreted more Kyn (3.00 ± 0.41 vs 1.94 ± 0.29 (mean ± SEM in mg/24 h; *n* = 8 vs 14; *P* = 0.044). Increased excretion of Kyn metabolites after Trp loading of rheumatoid patients was also attributed to drug (aspirin) therapy [61].

### Conclusions and comments

The present results have demonstrated the preferential docking (binding) of antidepressant drugs to tryptophan 2,3-dioxygenase, in accord with their ability to inhibit this major enzyme of Trp degradation and primary determinant of cerebral serotonin synthesis. While rapidly acting ketamine does not dock to TDO, we propose that its unique mode of action in MDD therapy is a novel one: that of targeting KMO to undermine neuronal dysfunction, thereby restoring serotonin homeostasis. Findings with the SSRI escitalopram [40] are also consistent with this proposed mechanism. Current evidence suggests that KMO overexpression occurs in MDD and its metabolic consequences override IDO induction by proinflammatory cytokines in patients with an inflamed profile. MDD pathology can therefore be attributed to serotonin deficiency with or without glutamatergic dysfunction, with Kyn pathway modulation being a shared feature. Future studies in MDD are likely to yield fruitful results by focusing on the Kyn pathway and, in particular, the roles of TDO and KMO and related novel therapeutic mechanisms.

## Supplementary Material

Supplementary Figures S1-S6

## Data Availability

The crystal structure of TDO from Xanthomonas campestris in complex with ferrous heme and Trp (Northeast Structural Genomics Target XcR13: PDB, ID, 2NW8), was taken from the protein data bank (http://www.rcsb.org/pdb). The IDO crystal structure selected for this study (PDB ID: 2D0T), that of the 4-phenylimidazole bound form of human indoleamine 2,3-dioxygenase, was taken from the protein data bank (PDB) (doi:10.2210/pdb2D0T/pdb). All other data are available directly from the authors.

## Abbreviations

3-HAA: 3-hydroxyanthranilic acid
3-HK: 3-hydroxykynurenine
5-HT: 5-hydroxytryptamine
5-HTP: 5-hydroxytryptophan
AA: anthranilic acid
ACMS: 2-amino-3-carboxymuconate-6-semialdehyde
AMS: 2-amino-3-muconate-6-semialdehyde
HBA: hydrogen bond acceptor
HBD: hydrogen bond donor
HBE: hydrogen bond energy
IDO: indoleamine 2,3-dioxygenase
KA: kynurenic acid
KMO: kynurenine monooxygenase
Kyn: kynurenine
NAD^+^: oxidised nicotinamide-adenine dinucleotide
PA: picolinic acid
QA: quinolinic acid
TDO: tryptophan 2,3-dioxygenase
Trp: tryptophan
XA: xanthurenic acid
