# Supplementary material for: Inflammation and serotonin deficiency in major depressive disorder: molecular docking of antidepressant and anti-inflammatory drugs to tryptophan and indoleamine 2,3-dioxygenases
Source: Biosci Rep. 2022 May 27;42(5):BSR20220426. doi: 10.1042/BSR20220426 (PMC9142829; doi:10.1042/BSR20220426)
Supplement: Supplementary Figures S1-S6 [file BSR-2022-0426_supp.pdf]

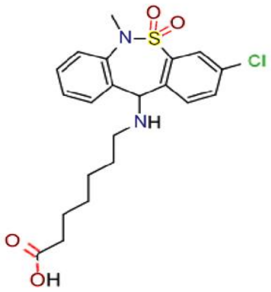
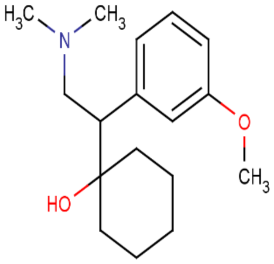
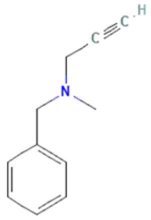

Tianeptine Venlafaxine Pargyline

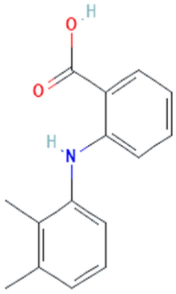
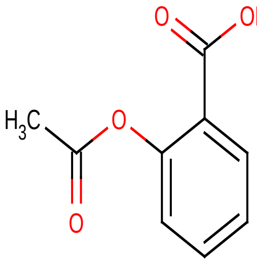
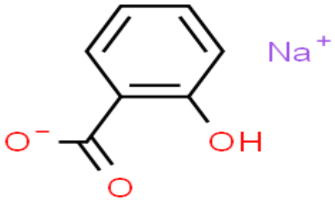

Mefenamic acid Acetylsalicylic acid Sodium salicylate

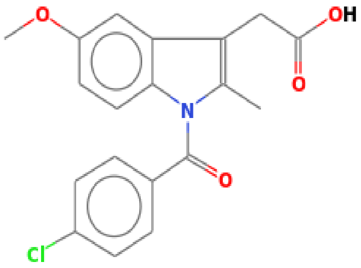
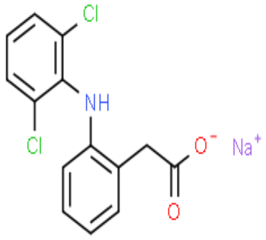
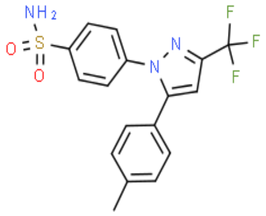

Indomethacin Diclofenac sodium Celecoxib

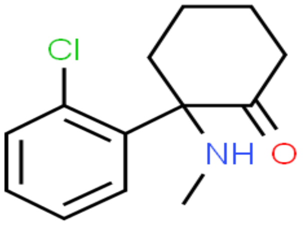

Ketamine

Figure S1. Structures of antidepressants and other drugs

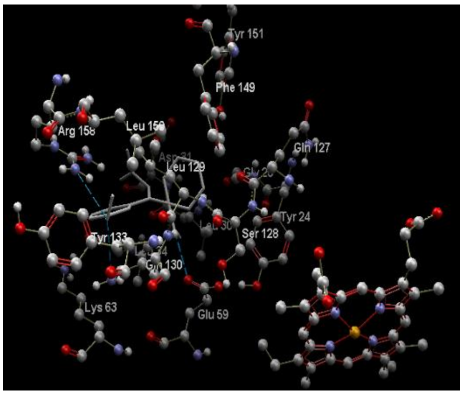

Venlafaxine-TDO

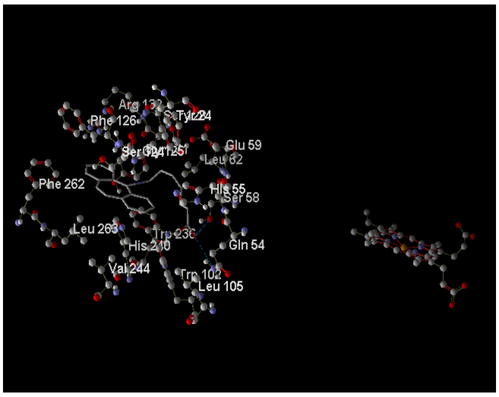

Tianeptine-TDO

…

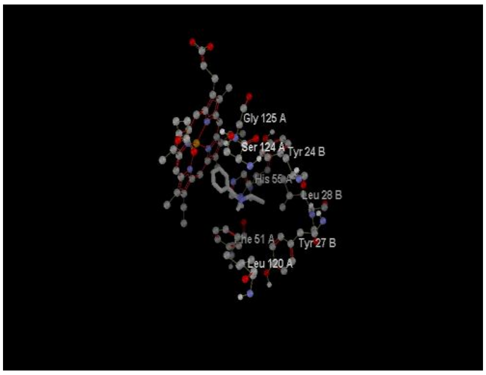

Pargyline-TDO

Figure S2. Molecular docking of venlafaxine, tianeptine and pargyline to Trp 2,3-dioxygenase (TDO)

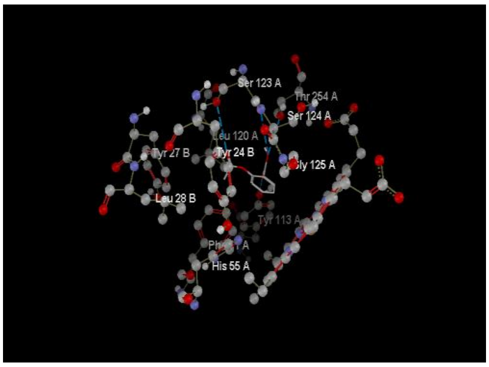

Acetylsalicylic acid-TDO

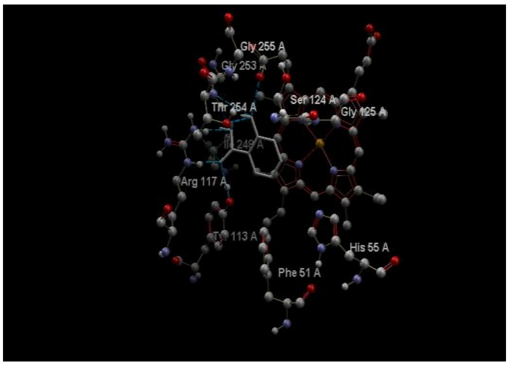

Sodium salicylate-TDO

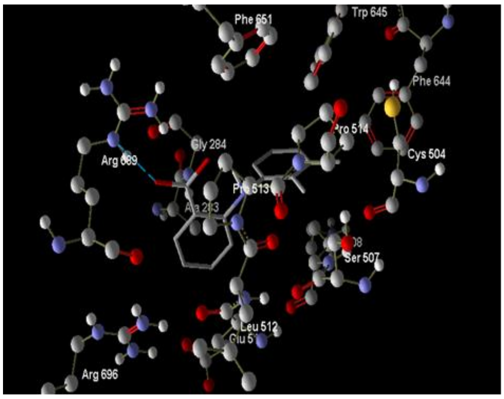

Mefenamic acid-TDO

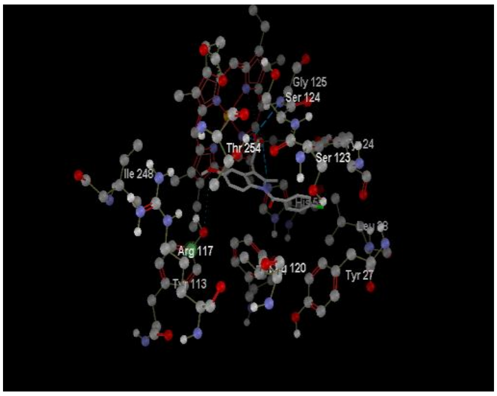

Indomethacin-TDO

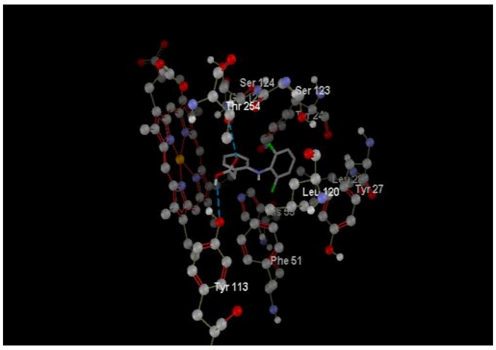

Diclofenac-TDO

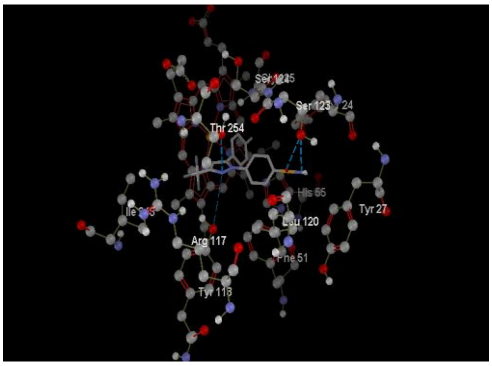

Celecoxib-TDO

Figure S3. Molecular docking of antiinflammatory drugs to tryptophan 2,3-dioxygenase (TDO)

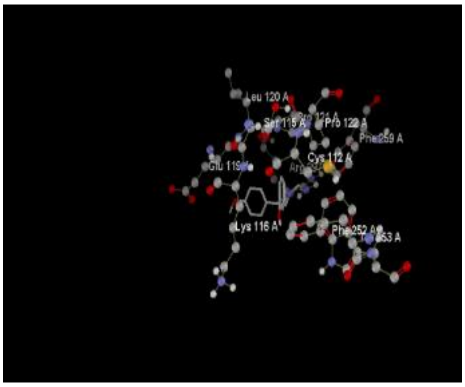

Venlafaxine-IDO

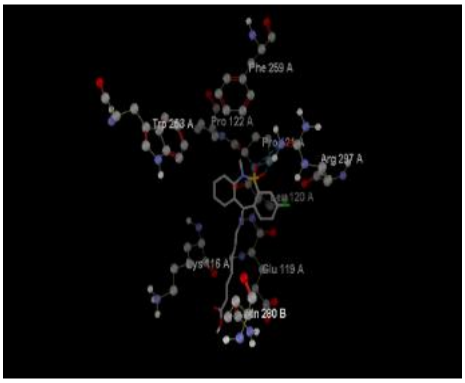

Tianeptine-IDO

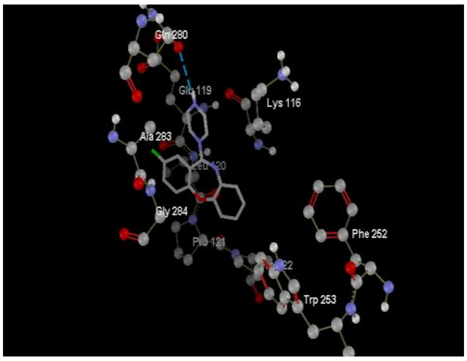

Amoxepine-IDO

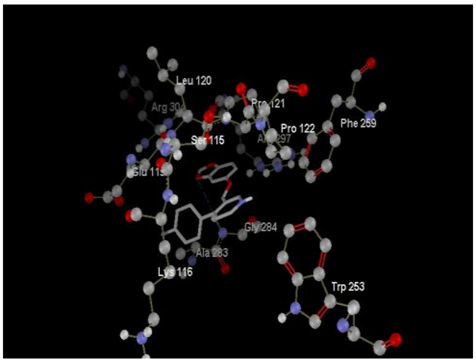

Paroxetine-IDO

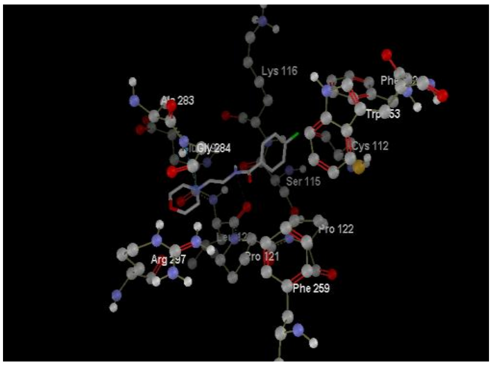

Moclobemide-IDO

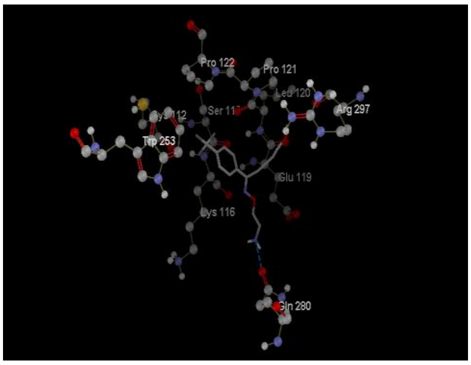

Fluvoxamine-IDO

Figure S4. Molecular docking of antidepressants to indoleamine 2,3-dioxygenase (IDO)

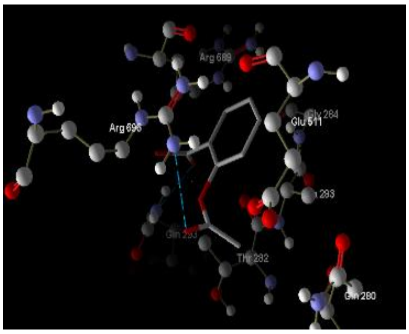

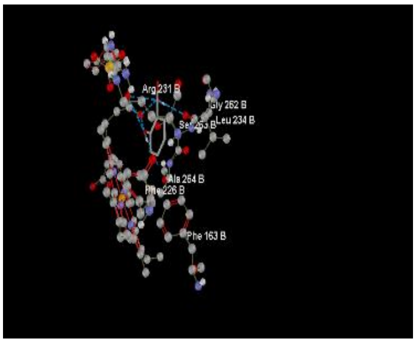

Acetylsalicylic acid-IDO

Sodium salicylate-IDO

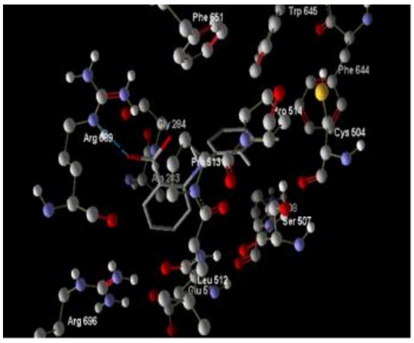

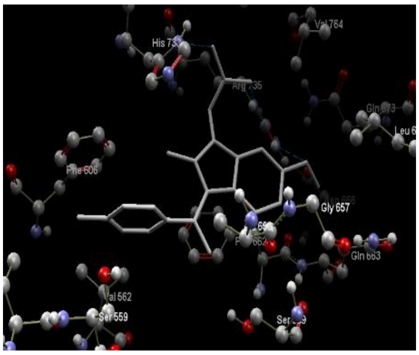

Mefenamic acid-IDO

Indomethacin-IDO

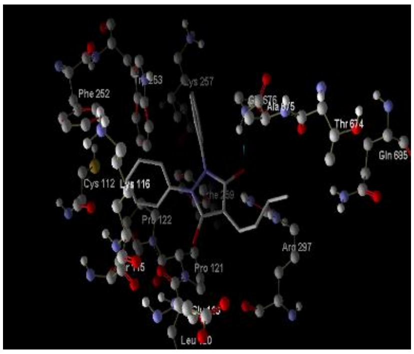

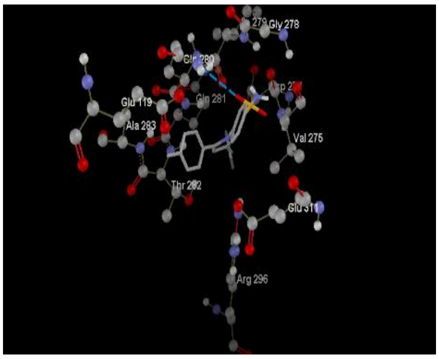

Diclofenac-IDO

Celecoxib-IDO

Figure S5. Molecular docking of antiinflammatory drugs to indoleamine 2,3-dioxygenase (IDO)

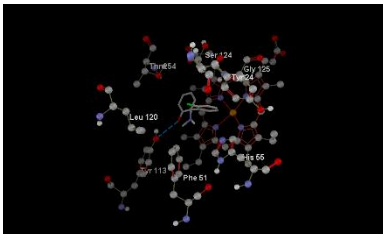

Ketamine-TDO

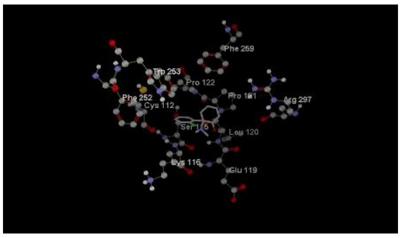

Ketamine-IDO

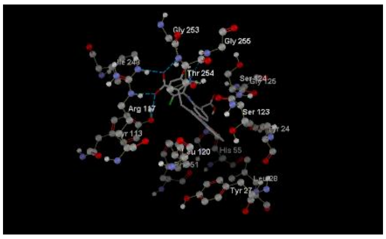

3-Hydroxydiclofenac-TDO

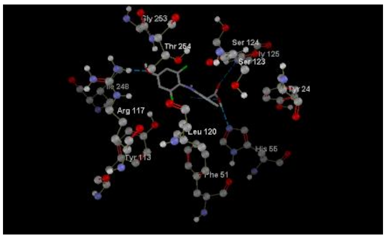

4-Hydroxydiclofenac-TDO

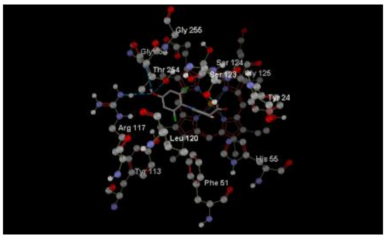

4,5-Dihydroxydiclofenac-TDO

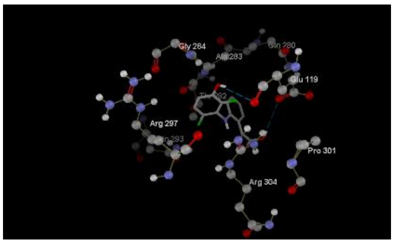

3-Hydroxydiclofenac-IDO

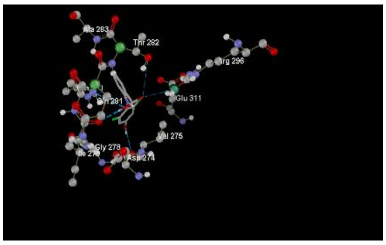

4-Hydroxydiclofenac-IDO

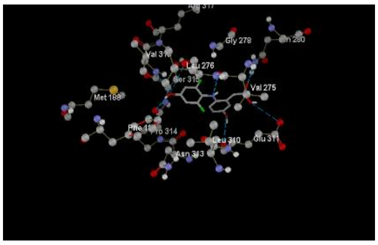

4,5-Dihydroxydiclofenac-IDO

Figure S6. Docking of ketamine and diclofenac metabolites to TDO and IDO

Fig. S1. Structures of antidepressant and other drugs

Fig. S2. Molecular docking of venlafaxine, tianeptine and pargyline to Trp 2,3-dioxygenase (TDO)
The TDO cofactor haem is presented in lines with red color. Amino acids in the active site are presented in ball and stick with element color and ligand is presented in thick lines with element color (where carbon is grey, oxygen is red, nitrogen is blue, sulphur is yellow and hydrogen is white). Blue lines represent the hydrogen bonds in between the ligand and the active site of TDO.
With venlafaxine, amino acid residues near the active site at docking were (Arg 158, Tyr 133, Gln 130, Leu159,129, Glu 59, Ser 128, Phe 149) and ligand binding amino acids were (Arg 158, Tyr 133, Gln 130, Glu 59).
With tianeptine, the corresponding amino acids were: (Phe 126, Gly 125, Phe 262, Ser 124, Trp 109, Arg 132) and (Arg 132, Ser 58).
With pargyline, the corresponding amino acids were (His 55, Leu 28, Phe 51, Ser 124, Gly 125) and (none).

Fig. S3. Molecular docking of antiinflammatory drugs to tryptophan 2,3-dioxygenase (TDO)
With acetylsalicylic acid, amino acid residues near the active site at docking were (Tyr 27, Phe 51, His 55, Leu 120, Tyr 24, Ser 123) and ligand binding amino acids were (Thr 254, Ser 124, Gly 125, Tyr 113).
With sodium salicylate, the corresponding amino acids were (Tyr 113, Thr 254, Phe51, His 55,Arg 117, Ser 124, Gly 255,Gly 253) and (Thr 254, Tyr 113, Arg 117, Gly 253, Gly255).
With Mefenamic acid, the corresponding amino acids were (Gly 125, Tyr 24, His, 55, Ser 123, Thr 254) and (Leu 120, Thr 254, Ser 124, Gly 125).
With indomethacin, the corresponding amino acids were (Ser124, Gly125, Ser123, Thr254, Arg117, Leu120,Tyr27) and (Gly125, Tyr24, Ser124)
With diclofenac, the corresponding amino acids were (Thr254, Leu120, Tyr 27, Phe51, Tyr113, Ser123,124) and (Tyr113, Thr254).
With celecoxib, the corresponding amino acids were (Thr254, Ser123, 124, His55, Tyr27, Leu120, Arg117, Ile248, Gly125) and (Ser124, Thr254, Ser123).

Fig. S4. Molecular docking of antidepressants to indoleamine 2,3-dioxygenase (IDO)
With venlafaxine, amino acid residues near the active site at docking were (Lys116, Phe 252, 253, Glu119, Leu120, Ser 115, Cys 112) an ligand binding amino acids were (none).
With tianeptine, the corresponding amino acids were: (Pro 122, Glu 119, Lys 116, Trp 253, Arg 297) and (Arg 297, Pro 121).
With amoxepine, the corresponding amino acids were (Gln280, Glu119, Lys116, Ala283, Gly284, Pro121) and (Glu119, Leu120).
With paroxetine, the corresponding amino acids were (Ser115, Lys 116, Gly 284, Leu120, Arg297, Pro 122) and (Gly284, Leu120, Lys116, Pro122).
With moclobemide, the corresponding amino acids were (Gly284, Ser 115, Arg297, Ala283, Leu120, Pro 122) and (Gly284).
With fluvoxamine, the corresponding amino acids were (Trp253, Ser117, Glu119, Lys116, Gln280, Leu 120, Lys253) and (Gln280, Lys116).

Fig. S5. Molecular docking of antiinflammatory drugs to indoleamine 2,3-dioxygenase (IDO)
With acetylsalicylic acid (aspirin), amino acid residues near the active site at docking were ((Gln 293, Thr 282, Glu 511, Arg 696, Arg 698, Gln 280) and ligand binding amino acids were (Arg 696, Gln 293, Glu 511, Gln 293).

With sodium salicylate, the corresponding amino acids were (Phe 266, Ala 264, Phe 163, Ser 263) and (Arg 23, Ala 264).
With mefenamic acid, the corresponding amino acids were (Gly 284, Ala 283, Pro 514, Cys 504, Ser 507, Leu 512, Arg 696) and (Arg 689).
With indomethacin, the corresponding amino acids were (Val 562, Ser 559, Gly 657, Phe 606) and (His733).
With diclofenac, the corresponding amino acids were (Pro 121, Pro 122, Trp 253, Lys 116, Phe 253, Lys 257) and (Arg 297, Gly 676).
With celecoxib, the corresponding amino acids were (Glu119, Thr282, Gln280, Arg296, Val276, Ala283) and (Gln 280, Gly 278).

Fig. S6. Molecular docking of ketamine and diclofenac metabolites to TDO and IDO
With R ketamine, amino acid residues near the TDO active site at docking were (Leu 120, Tyr113, Phe51, Thr254, His55, Tyr24, Gly125) and ligand binding amino acids were (Tyr113).
With ketamine HCl, the corresponding amino acids were (Leu120, Thr254, Ser123, 124, Phe51, His55, Tyr24) and (None).

With R ketamine docking to IDO, the corresponding amino acids were (Ser115, Cys112, Phe252, Trp253, Pro 161, Leu120, Glu119) and (None).
With ketamine HCl docking to IDO, the corresponding amino acids were (Leu120, Lys116, Ser115, Cys112, Trp 253, Glu119) and (None).

With 3-hydroxydiclofenac docking to TDO, amino acid residues near the active site at docking were (Arg117, Thr254, Ser124, Ser123, His55, Leu 120, Tyr 113) and legend binding amino acids were (Arg117, Thr254, Tyr113).
With 4-hydroxydiclofenac, the corresponding amino acids were (Thr254, Arg117, Leu120, Tyr113, Phe51) and (His55, Ser124, Arg117).
With 4,5-dihydroxydiclofenac, the corresponding amino acids were (Leu120, Tyr113, Phe51, Thr254,Ser123,124, Gly125) and (Thr254, Arg117, Gly255).

With 3-hydroxydiclofenac docking to IDO, amino acid residues near the active site at docking were (Arg297, Thr282, Ala283, Arg304, Glu119, Gln280) and ligand binding amino acids were (Glu119, Arg304, Gln280).
With 4-hydroxydiclofenac, the corresponding amino acids were (Thr282, Gln 280, 281, Gly278, Val275, Asp 275) and (Gln281, Thr282, Gly278, Asp274).
With 4,5-dihydroxydiclofenac, the corresponding amino acids were (Pro 134, Met 188, Val316, Ser315, Gly278, Glu311, Asn213) and (Gly278, Val316, Gln280).
